# Bedside Biliary Drainage without Fluoroscopy for Critically Ill Patients

**DOI:** 10.1155/2020/2850540

**Published:** 2020-06-15

**Authors:** Junbo Hong, Wei Zuo, Xiaodong Zhou, Xiaojiang Zhou, Guohua Li, Zhijian Liu, Anjiang Wang, Yin Zhu, Nonghua Lu, Youxiang Chen

**Affiliations:** ^1^Departments of Gastroenterology, The First Affiliated Hospital of Nanchang University, Nanchang, 330006 Jiangxi Province, China; ^2^Departments of Respiratory Medicine, The First Affiliated Hospital of Nanchang University, Nanchang, Jiangxi, China

## Abstract

**Background:**

Bedside biliary drainage by endoscopic retrograde cholangiopancreatography (ERCP) without fluoroscopy for critically ill patients in the intensive care unit (ICU) remains challenging for endoscopists. The present study was to evaluate the efficacy and safety of radiation-free ERCP for these patients.

**Methods:**

Consecutive ICU patients with severe pancreaticobiliary disorders who underwent bedside radiation-free ERCP were retrospectively analyzed.

**Results:**

Radiation-free ERCP was performed in 80 patients with acute physiology and chronic health evaluation (APACHE II) score of 24.1 ± 6.2. Cannulation was achieved in 75 (93.75%) patients. Biliary drainage was successfully conducted in 74 (92.5%) patients, including 54 (67.5%) and 20 (25.0%) cases of endoscopic retrograde biliary drainage (ERBD) and endoscopic nasobiliary drainage (ENBD), respectively. Adverse event (mild post-ERCP pancreatitis (PEP)) occurred only in 1 case. The 30-day mortality rate of these patients was 36.25% (29/80) and was much more higher in patients with ERBD in contrast to that of patients with ENBD, 40.7% (22/54) vs. 20% (4/20), OR = 2.750, 95%CI = 0.810 − 9.3405, *P* = 0.110. The APACHE II score in nonsurvivors was significantly higher than survivors, 27.6 ± 4.3 versus 22.2 ± 6.3, *P* = 0.009. The APACHE II score > 22 was an independent risk factor for mortality, 50% versus 10.7%, 95%CI = 2.148 − 31.569, *P* = 0.002.

**Conclusions:**

Radiation-free ERCP guided bedside biliary drainage is effective and safe for critically ill patients, and ENBD may be an optimal procedure due to a low mortality in these patients.

## 1. Introduction

ERCP is putatively effective and safe for managing pancreaticobiliary disorders [1, 2], and emergent ERCP with biliary drainage is needed for patients with severe acute cholangitis (SAC) who fail to respond to conservative treatment [3–5]. However, the risk will greatly increase when transporting critically ill patients to a facility without fluoroscopy for endoscopic drainage. Moreover, based on the fact that portable X-ray equipment is lacking in the majority of ICU, it is challenging for endoscopists to perform radiation-free ERCP for these patients.

Several assistant technologies have been incorporated into radiation-free ERCP in previous studies. Biliary drainage (ENBD and ERBD) is performed for critically ill patients in ICU under the guidance of extracorporeal ultrasound or intraductal ultrasound (IDUS) by checking the position of the guidewire after successful cannulation [6–9]. Furthermore, noncomplex choledocholithiasis has been successfully and safely extracted by radiation-free ERCP combined with cholangioscopy, endoscopic ultrasound (EUS), or IDUS [10–12]. Although the role of solitary radiation-free ERCP in emergent biliary decompression for critically ill patients has been demonstrated in a clinical study with small sample sizes [13], its efficacy and safety have not been systematically evaluated in a relatively large population of these patients to date. This goal was achieved in the present study.

## 2. Patients and Methods

### 2.1. Patients

We performed a retrospective study of all critically ill patients with multiple organ dysfunction syndromes (MODS) in ICU due to severe pancreaticobiliary disorders who underwent ERCP from February 2015 to December 2019 at the First Affiliated Hospital of Nanchang University (approximately 2500 ERCPs per year). The medical records, laboratory results, radiological studies, and endoscopy results were reviewed for all patients included in the study. All patients gave written informed consent for all procedures.

### 2.2. Perioperative Preparation

The results of routine blood tests, biochemical function (liver and renal function, serum myocardial enzymogram, electrolytes, and amylase), coagulation tests, procalcitonin levels, blood gas analysis, bedside echocardiography, and electrocardiograms were obtained. The etiologies and severity of pancreaticobiliary disorders were evaluated by clinical manifestations, laboratory, and radiological results of the patients. Initial fluid resuscitation, antibiotics, and vasoactive medications were administered; Patients were given fresh frozen plasma and/or vitamin K1 upon extension of more than 3 seconds of the upper limit of the prothrombin time and/or the international normalized ratio > 1.5; In addition, platelets were infused into thrombocytopenic patients when the platelet count was less than 20,000/*μ*L; Blood purification and mechanical ventilation were conducted when they were necessary [14]. Indications and contraindications for ERCP were evaluated by endoscopists and anesthesiologists before the procedure.

### 2.3. Evaluation of Cholangitis and Acute Pancreatitis

The diagnostic criteria and severity grading of acute cholangitis and acute pancreatitis were defined according to the Tokyo Guidelines 2018 and Revised Atlanta Classification, respectively [15, 16].

### 2.4. Calculation of APACHE II Scores

APACHE II score was calculated within 24 hours after admission as previously described [17].

### 2.5. ERCP Procedures

ERCP was performed by experienced endoscopists (more than 300 ERCPs per year) with a standard side-viewing duodenoscope. A cap-assisted forward-viewing endoscope was used for patients with surgically altered gastrointestinal anatomy. Wire-guided biliary cannulation with a sphincterotome was conducted in all patients, confirmed by the free placement of a guidewire and a free flow of bile. ENBD or ERBD was conducted under the guidance of the guidewire, after which was checked by free insertion of an ERCP catheter (at least 5 cm). Precut papillotomy was occasionally used for difficult cannulation determined by endoscopists. Prophylactic pancreatic stent would be placed if repeated unintentional access to the main pancreatic duct occurred defined by clear aspirate in the sphincterotome and syringe [10, 11, 18].

### 2.6. Outcome Evaluation

The primary outcome was a technical success, defined as the accomplishment of ERCP with successful biliary drainage (ERBD or ENBD) [19]. The secondary outcome was the prevalence of adverse events of the ERCP and 30-day mortality rate [13, 20].

### 2.7. Evaluation of Adverse Events of the ERCP

Adverse events (including PEP, bleeding, cholangitis, cholecystitis, perforation, and cardiopulmonary adverse events) were defined by the guideline of the American Society for Gastrointestinal Endoscopy Standards of Practice Committee [20].

### 2.8. Statistical Analysis

All statistical analyses were performed by the Statistical Package for Social Science software suite (version 17.0; SPSS, Inc., Chicago, IL, USA). The *χ*^2^-test or Fisher's exact test (for categorical data) and *t*-test (for numerical data) were used to estimate the significance of differences, which were described by 95% confidence interval (CI). Variables with *P* < 0.10 in the univariate analysis were entered as candidate risk factors in the multivariate forward stepwise logistic regression analysis. All tests were two-sided, and a *P* value of less than 0.05 was considered statistically significant.

## 3. Results

### 3.1. Baseline Characteristics

**Figure 1:**
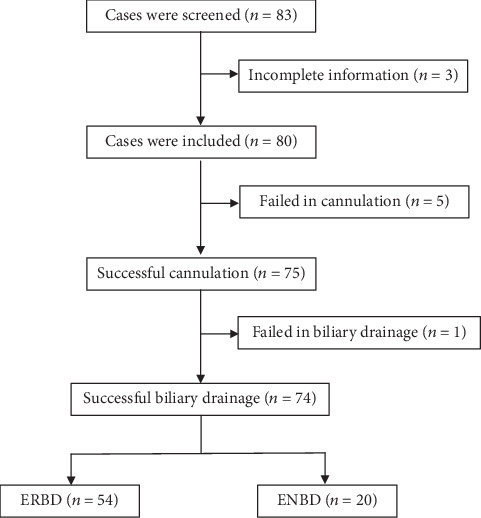
Flowchart of patients included in the study. ERBD: endoscopic retrograde biliary drainage; ENBD: endoscopic nasobiliary drainage.

A total of 83 patients were screened. Of these patients, 3 were excluded due to incomplete information. Ultimately, 80 patients (53 and 27 cases of male and female) with an average APACHE score 24.1 ± 6.2 were included in the study. No difference of APACHE II score was found between ERBD and ENBD, 23.6 ± 6.6 vs. 24.2 ± 6.1, *P* = 0.660 (Table 1). The average age of males and females was 68.4 ± 12.8 and 69.9 ± 11.4, respectively, *P* = 0.495 (Table 2 and Figure 1).

### 3.2. Indications for the ERCP

The indications for the ERCP were as follows: choledocholithiasis (*n* = 46, 57.5%), gallstone pancreatitis (*n* = 25, 31.25%) and others (*n* = 9, 11.25%), including perihilar cholangiocarcinoma (*n* = 2, 2.5%), distal cholangiocarcinoma (*n* = 1, 1.25%), carcinoma of the duodenal papilla (*n* = 2, 2.5%), pancreatic carcinoma (*n* = 1, 1.25%), hematobilia (*n* = 1, 1.25%), biliary leakage (*n* = 1, 1.25%), and traumatic hepatic rupture (*n* = 1, 1.25%) (Table 1).

### 3.3. The Severity Grading of Acute Cholangitis and Acute Pancreatitis

SAC occurred in 72 cases (90%), including all of the patients with choledocholithiasis (*n* = 46), majority of the gallstone pancreatitis (*n* = 20), and pancreaticobiliary carcinoma (*n* = 6). In addition, moderate and mild acute cholangitis occurred in 5 (6.25%) and 1 patient (1.25%), respectively. The prevalence of severe, moderate, and mild grading acute pancreatitis was 11 (13.75%), 9 (11.25%), and 5 (6.25%) cases, respectively (Table 2).

### 3.4. ERCP Procedures in the 80 Cases

Cannulation was achieved in 75 cases (93.75%), except for 5 cases due to edema of the duodenal papilla (*n* = 3), failure in finding the duodenal papilla (*n* = 1), and rigidity of duodenal papilla (*n* = 1). ERBD and ENBD was successfully conducted in 54 (67.5%) and 20 (25.0%) cases, respectively. Biliary drainage failed in one case with successful cannulation due to the incapability of implanting the guidewire in place. The nasobiliary catheter was occluded in 3 (15%) cases and recanalized by irrigation with normal saline. Endoscopic sphincterotomy (EST), endoscopic retrograde pancreatic drainage (ERPD), and stone extraction were performed in 2, 3, and 1 patients, respectively (Table 1).

### 3.5. Adverse Events

ERCP associated adverse event occurred only in one patient (mild PEP). The overall 30-day mortality rate of these patients was 36.25% (29/80), including multiple organ failure (*n* = 26) and myocardial infarction (*n* = 2) induced by SAC, and hemorrhage of necrotic pancreatitis (*n* = 1) (Table 1).

### 3.6. Risk Factors for 30-Day Mortality in 74 Cases

The 30-day mortality rate was much higher in patients with ERBD in contrast to that of patients with ENBD, 40.7% (22/54) vs. 20% (4/20), 95%CI = 0.810 − 9.340, *P* = 0.110. The APACHE II score in nonsurvivors was significantly higher than survivors, 27.6 ± 4.3 versus 22.2 ± 6.3, *P* = 0.009. The APACHE II score > 22 was an independent risk factor for mortality, 50% versus 10.7%, 95%CI = 2.148 − 31.569, *P* = 0.002 (Table 3).

## 4. Discussion

Little is known regarding the efficacy and safety of bedside ERCP without fluoroscopy and other assistant technologies for critically ill patients, which has been confirmed in the present study. In addition, our study demonstrated that the overall 30-day mortality rate of these patients was 36.25% and was much higher in patients with ERBD compared with ENBD. The APACHE II score > 22 was an independent risk factor for mortality.

Successful cannulation represents a fundamental step of ERCP operation; selective biliary cannulation can be achieved in more than 95% of cases in experienced hands [21, 22]. However, cannulation is still challenging for the patients with SAC possibly due to stone impaction or swelling of the papilla, and little is known regarding the cannulation rate in these patients. Qualified cannulation rates of 100% and 86% have been reported in two studies with 6 and 22 critically ill patients, respectively [6, 23]. The present study (*n* = 80) presented a successful cannulation rate of 93.75%, predominantly by a conventional method (precut sphincterotomy with needle-knife was performed only in two cases). Cannulation failed in five cases due to swelling, rigidity, or inconspicuousness of the papilla.

The role of biliary decompression including ERBD or ENBD by radiation-free ERCP without any assisted technology for critically ill patients in ICU has not been well elucidated. As mentioned previously, a nasobiliary catheter was successfully placed in 23 patients (88%) [13]. The present study demonstrated that biliary drainage was achieved in 74 cases (92.5%) and failed only in one case. In our knowledge, free insertion of a catheter (at least 5 cm) under the guidance of guidewire after cannulation is the basis of successful biliary drainage.

Optimization of the procedures (ERBD or ENBD) for biliary drainage remains a tough issue. Although three clinical trials have demonstrated that biliary decompression by nasobiliary catheter is as effective and safe as indwelling stent for patients with SAC, stent obstruction by purulent and sticky biliary sludge may still occur [24–26]. Furthermore, the mortality rate is higher in patients with ERBD compared to ENBD (12% versus 2.5%) [24]. We addressed ERBD and ENBD in 54 (67.5%) and 20 (25.0%) cases, respectively, and ERBD was also associated with a higher mortality rate by contrast with ENBD (40.7% vs. 20%). In addition, the volume of drainage can be dynamically monitored and the patency of nasobiliary tube can be ensured by constant irrigation with normal saline. Thus, ENBD might be an optimal procedure for patients with SAC.

APACHE II score classification system has been putatively applied for measuring the severity of illness for critically ill patients [17]. Mortality of the critically ill surgical patients in ICU significantly increases with a higher APACHE II score [27], and the odds ratios for the first 28-day mortality of ICU patients is 2.56 (95% CI, 2.26 to 2.92) for the APACHE II score ≥ 20 [28]. In addition, survivors among the patients with septic shock due to SAC have lower APACHE II scores compared to nonsurvivors (22 versus 28, *P* < 0.001) [29]. Furthermore, patients with severe pancreaticobiliary diseases have 100% mortality when the APACHE II scores ≥ 20 [22]. We also demonstrated that the APACHE II score in nonsurvivors was significantly higher than survivors, and the APACHE II score > 22 was an independent risk factor for mortality.

The most common complications for ERCP are PEP (3%–14.7%), cholangitis (0.5%–3%), hemorrhage (0.3%–2%), gastrointestinal perforation (0.08%–0.6%), and cardiopulmonary complications (0.07%–5.3%) [19]. A previous study has revealed complication rates of 5% and 0 in patients undergo ENBD (*n* = 40) and ERBD (*n* = 34) by radiation-based ERCP for acute suppurative cholangitis [23]. Furthermore, no ERCP associated complications occurred in critically ill patients (*n* = 26) who underwent ERCP without fluoroscopic guidance [13]. We also demonstrated that adverse event (mild PEP) occurred only in 1 patient, with an overall rate of 1.25% (1/80). Thus, urgent biliary decompression by radiation-free ERCP without any assisted technology is safe for critically ill patients in ICU.

Certain limitations were available in this study. The efficacy and safety of ERBD and ENBD evaluated and compared in this study were retrospectively and not randomly. In addition, the risk factors (the level of total bilirubin, lactic acid, and leukocyte count, etc.) for 30-day mortality were not systematically evaluated due to a limited sample size.

## 5. Conclusions

In summary, radiation-free ERCP guided bedside biliary drainage is feasible for critically ill patients due to its superior efficacy and safety, ENBD may be an optimal procedure. Prospective and randomized studies with large populations are needed to verify these results.

## Figures and Tables

**Table 1:** ERCP indications, procedure, and adverse events in 80 patients (*n*, %).

Indications	
Choledocholithiasis	46 (57.5)
Gallstone pancreatitis	25 (31.25)
Perihilar cholangiocarcinoma	2 (2.5)
Distal cholangiocarcinoma	1 (1.25)
Carcinoma of duodenal papilla	2 (2.5)
Pancreatic carcinoma	1 (1.25)
Hematobilia	1 (1.25)
Biliary leakage	1 (1.25)
Traumatic hepatic rupture	1 (1.25)
Procedure	
Cannulation	
Succeed	75 (93.75)
Failed^a^	5 (6.25)
ERBD	54 (67.5)
ENBD	20 (25)
EST	2 (2.5)
ERPD	3 (3.75)
Stone extraction	1 (1.25)
Adverse events	
Mild acute pancreatitis	1 (1.25)
Mortality^b^	29 (36.25)
APACHE II score	
ERBD^c^	24.2 ± 6.1
ENBD	23.6 ± 6.6

EST: endoscopic sphincterotomy; ERPD: endoscopic retrograde pancreatic drainage. ^a^Including failed in finding the duodenal papilla (*n* = 1), edema of the duodenal papilla (*n* = 3), and rigidity of duodenal papilla (*n* = 1); ^b^including multiple organ failure (*n* = 26), myocardial infarction (*n* = 2), and hemorrhage of necrotic pancreatitis (*n* = 1); ^c^no difference between ERBD and ENBD, *t* = −0.381, *P* = 0.660.

**Table 2:** Demographics, the prevalence of acute cholangitis, and pancreatitis in 80 patients.

Demographics	*n* (%)
Sex	
Male	53 (66.25)
Female	27 (33.75)
Age	
Male	68.4 ± 12.8
Female	69.9 ± 11.4
Acute cholangitis	
Severe^d^	72 (90)
Moderate	5 (6.25)
Mild	1 (1.25)
Acute pancreatitis	
Severe	11 (13.75)
Moderate	9 (11.25)
Mild	5 (6.25)
Causes of ICU admission	
MODS	80 (100)
APACHE II score	24.1 ± 6.2

^d^Including choledocholithiasis (*n* = 46), gallstone pancreatitis (*n* = 20), and pancreaticobiliary carcinoma (*n* = 6). APACHE: acute physiology and chronic health evaluation. MODS: multiple organ dysfunction syndromes.

**Table 3:** Risk factors for mortality in 74 cases (*n*, %).

	Nonsurvivors	Survivors	Univariate analysis		Multivariate analysis	
			95% CI	*P*	95% CI	*P*
APACHE II^e^						
>22	23 (50)	23 (50)	2.205-31.500	0.001	2.148-31.569	0.002
≤22	3 (10.7)	25 (89.3)				
Biliary drainage						
ERBD	4 (20)	16 (80)	0.810-9.3405	0.110	0.101-1.380	0.14
ENBD	22 (40.7)	32 (59.3)				

^e^The APACHE II score in nonsurvivors was significantly higher than survivors, 27.6 ± 4.3 versus 22.2 ± 6.3, *P* = 0.009.

## Data Availability

The data used to support the findings of this study are available from the corresponding author upon request.

## Abbreviations

ERCP: Endoscopic retrograde cholangiopancreatography
EST: Endoscopic sphincterotomy
EPBD: Endoscopic papillary balloon dilation
ENBD: Endoscopic nasobiliary drainage
ERPD: Endoscopic retrograde pancreatic drainage
ICU: Intensive care unit
APACHE II: Acute physiology and chronic health evaluation
PEP: Post-ERCP pancreatitis
SAC: Severe acute cholangitis
IDUS: Intraductal ultrasound
CI: Confidence interval
MODS: Multiple organ dysfunction syndromes.
